# Investigation of the Pharmacological Properties of *Lepidagathis hyalina* Nees through Experimental Approaches

**DOI:** 10.3390/life11030180

**Published:** 2021-02-25

**Authors:** Fowzul Islam Fahad, Niloy Barua, Md. Shafiqul Islam, Syed Al Jawad Sayem, Koushik Barua, Mohammad Jamir Uddin, Md. Nazim Uddin Chy, Md. Adnan, Mohammad Nazmul Islam, Mohammed Aktar Sayeed, Talha Bin Emran, Jesus Simal-Gandara, Ester Pagano, Raffaele Capasso

**Affiliations:** 1 Department of Pharmacy, International Islamic University Chittagong, Chittagong 4318, Bangladesh; fowzul.fahad19@gmail.com (F.I.F.); niloybaruaniloy@gmail.com (N.B.); shafiqulislam.pharmacy@gmail.com (M.S.I.); aljawadsayem@gmail.com (S.A.J.S.); antukoushik@gmail.com (K.B.); jamiruddin3775@gmail.com (M.J.U.); nazim@gustoarg.org (M.N.U.C.); mdadnan@kangwon.ac.kr (M.A.); nazmul@iiuc.ac.bd (M.N.I.); 2 Department of Bio-Health Convergence, College of Biomedical Science, Kangwon National University, Chuncheon 24341, Korea; 3 Department of Pharmacy, BGC Trust University Bangladesh, Chittagong 4381, Bangladesh; 4 Nutrition and Bromatology Group, Department of Analytical and Food Chemistry, Faculty of Food Science and Technology, Ourense Campus—University of Vigo, E32004 Ourense, Spain; 5 Department of Pharmacy, University of Naples Federico II, 80131 Naples, Italy; ester.pagano@unina.it; 6 Department of Agricultural Sciences, University of Naples Federico II, 80055 Portici, Italy

**Keywords:** *Lepidagathis hyalina*, antioxidants, cytotoxic, thrombolytic, anxiolytic, antidepressant

## Abstract

*Lepidagathis hyalina* Nees is used locally in Ayurvedic medicine to treat coughs and cardiovascular diseases. This study explored its pharmacological potential through in vivo and in vitro approaches for the metabolites extracted (methanolic) from the stems of *L. hyalina*. A qualitative phytochemical analysis revealed the presence of numerous secondary metabolites. The methanol extract of *L. hyalina* stems (MELHS) showed a strong antioxidative activity in the 1,1-diphenyl-2-picrylhydrazyl (DPPH) and reducing power assays, and in the quantitative (phenolic and flavonoid) assay. Clot lysis and brine shrimp lethality bioassays were applied to investigate the thrombolytic and cytotoxic activities, respectively. MELHS exhibited an expressive percentage of clot lysis (33.98%) with a moderately toxic (115.11 μg/mL) effect. The in vivo anxiolytic activity was studied by an elevated plus maze test, whereas the antidepressant activity was examined by a tail suspension test and forced swimming test. During the anxiolytic evaluation, MELHS exhibited a significant dose-dependent reduction of anxiety, in which the 400 mg/kg dose of the extract showed 78.77 ± 4.42% time spent in the open arm in the elevated plus maze test. In addition, MELHS demonstrated dose-dependent and significant activities in the tail suspension test and forced swimming test, whereas the 400 mg/kg dose of the extract showed 87.67 ± 6.40% and 83.33 ± 6.39% inhibition of immobile time, respectively. Therefore, the current study suggests that *L. hyalina* could be a potential source of anti-oxidative, cytotoxic, thrombolytic, anxiolytic, and antidepressant agents. Further study is needed to determine the mechanism behind the bioactivities.

## 1. Introduction

Free radicals such as reactive oxygen species (ROS) and reactive nitrogen species (RNS) are byproducts of several physiological and biological processes leading to oxidative stress in the human body. Biomolecules (e.g., DNA, protein, lipids, etc.) are damaged by the overproduction of such free radicals, which play an important role in generating numerous chronic diseases [1,2]. Several types of atherothrombotic diseases, such as myocardial or cerebral infarction, occur due to thrombosis. When a homeostatic imbalance occurs in an artery, thrombus or blood clots are formed, which block the vascular organs and produce fatal signs that ultimately cause death [3].

According to the World Health Organization (WHO), nearly 450 million people suffer from anxiety and multiple depressive disorders, which make up around 12.3% of the global burden of diseases [4,5]. Depression is the predominant disorder associated with forms of emotional and cognitive disablement, such as impaired thinking and activity, energy loss, apathy, etc. This psychiatric illness increases the risk of mortality (every year, 10 to 20 million people attempt suicide) [6]. In addition, anxiety, which is another psychiatric illness, is the sixth major contributor to non-fatal health suffering worldwide, according to the WHO [7,8,9].

Many genetic, environmental, psychological, and biological factors are involved in the progression of psychiatric disorders. Therefore, chronic pain and inflammation are linked to the onset of depression and the development of anxiety. The most daunting consideration with these agents is concern about their safety because of their unwanted side effects [10,11,12,13,14,15,16,17]. Current research has focused on developing potent and safer molecules for healing numerous disorders. Therefore, a drug which can generate activity against psychiatric disorders, scavenge ROS, and exhibit thrombolytic activities with a favorable safety profile, may be the best choice for both psychiatric and cardiovascular disorders. Interestingly, nature is the most prominent source of secondary metabolites that are helping in the discovery of new drug molecules with potency, efficacy, and favorable safety profiles. Numerous essential bioactive molecules (such as phenolics, saponins, terpenoids, alkaloids, etc.) of medicinal plants have explored multifaceted pharmacological targets that may be observed to be significant when compared with synthetically developed drugs [18,19].

*Lepidagathis hyalina* Ness is a subtropical habituated wild herb plant of the Acanthaceae family, known as curved Lepidagathis. It has been prescribed in Ayurvedic medicine for the treatment of coughs and cardiovascular diseases. It is mainly found in South Asian countries. In Bangladesh, it is widely available in hill tract areas. A previously reported article on this plant reported that a bioactive compound, named triterpenoid saponin (3-β-*O*-[α-l-rhamnopyranosyl(1→4)*O*-β-d-glucopyranosyl]16-α-hydroxy-olean-12-en(13)-28-oic acid), had been isolated from the leaf of this plant. In addition, a previous pharmacological study of this plant has shown that it provides good antimicrobial activity against pathogenic bacteria and fungi [20,21]. 

Until now, there have been very few scientific reports regarding this plant (*L. hyalina*), despite the fact that it is traditionally considered important. Therefore, we aim to investigate the quantitative phytoconstituents and scrutinize the antioxidant, cytotoxic, thrombolytic, and neuropharmacological activities of a methanol extract of *L. hyalina* stems. 

## 2. Materials and Methods

### 2.1. Chemicals and Equipment

Methanol, Folin–Ciocalteu reagent (FCR), potassium ferricyanide, sodium carbonate, aluminum chloride, potassium acetate, hydrochloric acid, and sulfuric acid were obtained from Merck (KGaA, Darmstadt, Germany). Quercetin, trichloro-acetic acid (TCA), sodium acetate, gallic acid, ferric chloride, and 1,1-diphenyl-2-picrylhydrazyl (DPPH) were purchased from Sigma Chemical Co. (St. Louis, MO, USA). Diclofenac sodium and diazepam were obtained from Square Pharmaceutical Ltd. Bangladesh. Vincristine sulfate (1 mg/vial) and lyophilized streptokinase vial (1,500,000 IU) were purchased from Beacon Pharmaceutical Ltd. Bangladesh (Bhaluka, Mymensingh, Bangladesh). The absorbance of the experiment was recorded using an Ultra-Violet-Vis spectrophotometer (UVmini-1240, Shimadzu, Japan). Several chemicals of analytical reagent grade with specified references were used in this research. 

### 2.2. Plant Materials 

In March 2019, stems of *L. hyalina* Ness were collected in a fresh condition from the Golden Temple Hill tract area, Bandarban, Chittagong-4600, Bangladesh. The sample was authenticated by Shaikh Bokhtear Uddin, Professor and Taxonomist, Department of Botany, University of Chittagong, Chittagong-4331, Bangladesh. The plant sample was then confirmed and identified by Mohammed Aktar Sayeed, Professor, Department of Pharmacy, International Islamic University Chittagong, Chittagong-4318, Bangladesh. 

### 2.3. Preparation of the Methanolic Crude Extract

The collected stems of *L. hyalina* were dried for 14 days in shade. Dried stems were then ground into a coarse powder through an automated grinder and dried in a mechanical drier at 60–70 °C. The fine stem powder was submerged in an adequate volume of methanol for ten days at room temperature, and the solution was then shaken vigorously. After this process, the solution was filtered using a rotary evaporator and dried at a temperature of 40–50 °C in a water bath. A sticky semi-solid with a deep green color was formed. This was preserved in a refrigerator and used as methanol extract. 

### 2.4. Standardization and Quality Control of the Extract

The methanol extract of *L. hyalina* stems (MELHS) was standardized and quality and physiochemical control of the crude extract was undertaken to ensure the safety and acute toxicity study of animal models [22].

### 2.5. Phytochemical Screening 

Preliminary qualitative phytochemical analysis of MELHS was performed by the standard method for the determination of phytochemicals such as carbohydrates, alkaloids, protein, quinones, saponins, tannin, starch, phenols, flavonoids, unsaturated sterols and triterpene, cardiac glycoside, and coumarins [23,24].

### 2.6. In Vitro Antioxidant Activity

#### 2.6.1. DPPH Free Radical Scavenging Assay

Free radical scavenging of MELHS was conducted according to the method described by Barca et al. [25]. About 3 mL of 0.004% DPPH solution was added with various concentrations (15.625 to 500 µg/mL) of crude extract, while a methanol plus DPPH solution was used as a negative control. The mixed solutions were then incubated for half an hour at a 30 °C temperature in a darkened room. A UV spectrophotometer was used to measure the absorbance at 517 nm. The reduction of absorbance with a high concentration implies effective radical scavenging activity. As a reference standard, ascorbic acid was applied [26]. The inhibition percentage of free radicals was measured by the following Equation (1):

(1) $$\%\;\mathrm{Scavenging}\;\mathrm{activity}=\frac{\mathrm{Ac}-\mathrm{As}}{\mathrm{Ac}}\times 100,$$

where Ac is the absorbance of the control and As is the absorbance of the sample.

#### 2.6.2. Power Reduction Assay

Reduction of the power capacity of MELHS was evaluated by following the method of Oyaizu [27]. About 1 mL of serially diluted concentration (31.25 to 500 µg/mL) was mixed with 2.5 mL of phosphate buffer (0.2 M, pH 6.6) and potassium ferricyanide (1% *w*/*v*). The mixed solution was incubated for 20 min at a 50 °C temperature to complete the reaction. After incubation, 2.5 mL of trichloroacetic acid (10%) was added and the whole mixture was centrifuged for 10 min at 3000 RPM. After that, the supernatant solution was dispelled and 0.5 mL of ferric chloride (0.1% *w*/*v*) was added to the solution with 2.5 mL of distilled water gradually mixed in. Therefore, the absorbance of the mixer was investigated on a UV spectrophotometer at 700 nm. A phosphate buffer was used as a blank solution, while ascorbic acid was used as a reference standard.

#### 2.6.3. Total Phenolic Content

The total phenolic content (TPC) of MELHS was investigated by the method of Singleton et al. as an oxidizing agent, and Folin–Ciocalteu reagent (FCR) was applied [28]. Two and a half milliliters of sodium carbonate (20%) was mixed with 2.5 mL of FCR, which was diluted 10 times with water. Then, a 500 µg/mL extract was combined with the mixture. After that, distilled water was added to the mixed solution to make up a 10 mL solution and kept in incubation for 20 min at a 25 °C temperature to react effectively. The absorbance of the sample was taken at 760 nm on the UV spectrophotometer. TPC was calculated from a calibration curve using a standard gallic acid solution of several concentrations, whereas the absorbance value plotted against concentrations and the results was assessed in mg gallic acid equivalent concentrations.

#### 2.6.4. Total Flavonoid Content

The total flavonoid content of MELHS was determined by the colorimetric method described by Chang et al., whereas quercetin was used as a reference standard [29]. About 100 μL of aluminum chloride (10%) and 1.5 mL of methanol were mixed with a 500 µg/mL extract solution. Afterward, 100 μL of potassium acetate (1 M) and 2.8 mL of distilled water were mixed into the solution. The mixed solution was incubated for half an hour at room temperature (37 °C) so that the mixture reaction could be completed. A UV spectrophotometer was used to measure the absorbance of the mixed solution at 415 nm against the blank solution, which contained all the reagents except the extract. The calibration curve was developed by employing several quercetin concentrations and the total flavonoid content was assessed in mg/g of quercetin equivalent. 

### 2.7. Brine Shrimp Lethality Bioassay

Brine shrimp eggs (*Artemia salina* leach) were used as a test organism to inspect the toxic properties of the extract. Shrimp eggs were hatched in artificial seawater, which was developed using sea salt (38 g/L), with NaOH (1N) added to adjust the pH to 8.5, and then placed at room temperature (37 °C) with a constant oxygen supply. After that, the hatched eggs were allowed to mature for 48 h and shrimp larvae known as nauplii were obtained. The procedure of the cytotoxic bioassay was performed according to the method of Meyer et al. [30]. The plant extract was dissolved in DMSO (5 mg/mL) to make a test sample with artificial seawater. Through serial dilution, several concentrations (31.25 to 1000 µ/mL) were obtained. Vincristine sulfate was used as a positive control, also using serial dilution to obtain different concentrations from 0.125 to 10 µg/mL, as in the preceding method. Ten living nauplii were added to the experimental vials and control vials and incubated for one day under light at room temperature. An amplifying glass was used to inspect all vials so that the numbers of living nauplii could be calculated and recorded for each vial. The mortality percentage of nauplii was calculated using Equation (2):

(2) $$\mathrm{Percentage}\;\left(\%\right)\;\mathrm{of}\;\mathrm{mortality}=\frac{N_{0}-N_{1}}{N_{0}}\times 100,$$

where $N_{0}$ is the number of nauplii taken and $N_{1}$ is the number of nauplii alive.

### 2.8. In Vitro Thrombolytic Activity

The thrombolytic activity of MELHS was explored using the method previously described by Prasad et al. [31]. As a stock solution, a vial of lyophilized streptokinase (1,500,000 I.U.) was diluted with 5 mL of sterile distilled water. Five milliliters of venous blood was drawn from six volunteers who were healthy and had no history of anticoagulant therapy. After that, the blood was distributed among six different pre-weighed sterile microcentrifuge tubes in the amount of 0.5 mL/tube and incubated at 37 °C for 45 min. After clot enhancement, the serum was carefully removed without disturbing the clot formation, which was weighed once again. A volume of 100 µL (10 mg/mL) of plant extract was added to each tube. Then, distilled water (100 µL) and streptokinase (100 µL) were separately added to the negative (non-thrombolytic) and positive control group, respectively, whereas water was used as a negative control. After this, all the tubes were incubated again for one and a half hours at 37 °C. Finally, the released fluid was cleaned and all tubes were weighed once again to calculate the weight difference after clot disruption. The percentage of clot lysis was calculated using Formula (3):

% clot lysis = (weight of clot after removing the fluid/weight of clot) × 100.(3)

This human-related experiment was conducted according to the ethical standards laid down in the 1964 Declaration of Helsinki. This study protocol was approved by the Department of Pharmacy, International Islamic University Chittagong, Chittagong, Bangladesh (ref. number: IIUC/PHARM-AEC-150/20-2019).

### 2.9. In Vivo Pharmacological Activity

#### 2.9.1. Experimental Animals and Ethical Statement

All animal experiments were carried out at the Department of Pharmacy, International Islamic University Chittagong, Chittagong-4318, Bangladesh. Male and female Swiss albino mice weighing around 25–30 g were purchased from Jahangirnagar University, Savar-1342, Bangladesh. The animals were housed in polypropylene cages (120 × 30 × 30 cm) and kept in standard conditions (25 ± 2 °C, 55–60% relative humidity, and 12 h light/dark circle) with a supply of water and food pellets. All mice were acclimated for 14 days to adapt to laboratory conditions before the experiments were started. Every effort was made to minimize the suffering of the animals. At the end of the observation period, all mice were euthanized using diethyl ether anesthesia. All the protocols followed in this experiment were approved by the Institutional Animal Ethical Committee, Department of Pharmacy, International Islamic University Chittagong, Chittagong-4318, Bangladesh, according to government guidelines under the reference number IIUC/PHARM-AEC-150/20-2019 [32]. All the sections of this report adhere to the “Animal Research: Reporting of In Vivo Experiments” guidelines for reporting animal research. The “Principles of the Laboratory Animal Care” (NIH publication no. 85-23, revised 1985) and “National Animal Care Laws” were strictly followed during the handling of the animals in this study.

#### 2.9.2. Experimental Design

The experimental animals were randomly separated into four different groups (standard, control, and test groups), with six Swiss albino mice included in each group. MELHS was administered to the test groups at two different dosages (200 and 400 mg/kg, b.w, p.o., respectively) and the vehicle (1% Tween-80 in water) was administered to the control group at a dosage of 10 mL/kg (p.o.). Standard Fluoxetine (20 mg/kg) was given intraperitoneally for the tail suspension test (TST) and forced swimming test (FST), while the drug diazepam (1 mg/kg, b.w, i.p.) was treated as standard for the elevated plus maze test (EPM). The plant extract doses and vehicle were received half an hour before the experiment and the reference drugs 15 min before the experiment.

#### 2.9.3. Acute Oral Toxicity Test

The acute oral toxicity test was carried out according to the “OECD Guidelines” [33]. Several doses of methanol extract (500, 1000, 2000, and 4000 mg/kg) were given orally to six Swiss albino mice who had fasted for 18 h before the administration of the doses. The mice were investigated individually for unusual signs, including allergic syndrome (itching, skin rash, swelling, etc.) and mortality within 72 h.

#### 2.9.4. Anxiolytic Activity

##### Elevated Plus Maze Test

The elevated plus maze test was used to screen the activity of the anxiolytic properties of MELHS in mice [34]. The experiment was carried out in a sound-attenuated room. The equipment used in this experiment consisted of four arms, with two open arms (5 × 10 cm) and two closed arms (5 × 10 × 15 cm) combined on a focal point platform (5 × 5 cm) in a plus sign configuration. All the groups of mice were administered different doses, as described in Section 2.9.2. Half an hour after the administration of the doses, a single animal from one of the four different groups was placed at the pivotal point of the platform facing the closed arm and allowed to roam for five minutes. An animal which placed all four of its paws onto any arm was recorded as having entered the arm. The total number of entries in the different types of arm (closed or open) and the times spent there were recorded. At the end of the experiment, the mice were euthanized using diethyl ether anesthesia. The percentage of entries into the open arm was calculated using Formula (4):

(4) $$\%\;\mathrm{of}\;\mathrm{entries}\;\mathrm{in}\;\mathrm{open}\;\mathrm{arm}\;=\frac{\mathrm{Number}\;\mathrm{of}\;\mathrm{entries}\;\mathrm{in}\;\mathrm{open}\;\mathrm{arm}}{\mathrm{Number}\;\mathrm{of}\;\mathrm{entries}\;\mathrm{open}\;\mathrm{arm}+\mathrm{Number}\;\mathrm{of}\;\mathrm{entries}\;\mathrm{close}\;\mathrm{arm}}\times 100.$$

#### 2.9.5. Antidepressant Activity

##### Tail Suspension Test

The tail suspension test was carried out following the method of Steru et al. [35]. All groups of mice were given different doses, as described in Section 2.9.2. Mice from each of the groups were individually suspended for six minutes in a box (25 × 25 × 30 cm), held by adhesive tape attached to the ends of their tails. Their total time of immobility was documented during the final four minutes of the six-minute suspension. The experiment was carried out with minimal ambient noise. 

##### Forced Swimming Test 

The forced swimming test was conducted using the method of Porsolt et al. [36] to investigate the in vivo antidepressant activity. The experiment was divided into two sessions: The first was carried out the day before the second to enable the animals to adapt to the environment. Immobility was created by placing the mice in a transparent glass tank (25 × 15 × 25 cm) filled with 15 cm of water at room temperature. Control, standard, and test group mice were treated with doses as described in Section 2.9.2. Each mouse was placed in the tank half an hour after the dose had been administered, and was allowed to swim around for six minutes. The first two minutes were regarded as a preliminary adjustment time. The final four minutes were used for measuring immobility.

### 2.10. Statistical Analysis

SPSS software was used for data analysis and GraphPad Prism version 6.0 was used to draw the figures. The results are presented as the mean ± SEM (standard error mean), and *p* values of less than 0.05, 0.01, and 0.001 were considered statistically significant (Dunnett’s multiple comparison test). 

## 3. Results

### 3.1. Qualitative Phytochemical Investigation

Qualitative phytochemical experiments were conducted for the *L. hyalina* stem extract to expose the presence of alkaloid, carbohydrate, reducing sugar, flavonoid, saponin, tannin, unsaturated sterol, triterpene, phenol, quinone, cardiac glycoside, and coumarin. The output of the various biochemical tests is summarized in Table 1 below.

### 3.2. Antioxidant Activity

#### 3.2.1. DPPH Scavenging Activity

**Figure 1:**
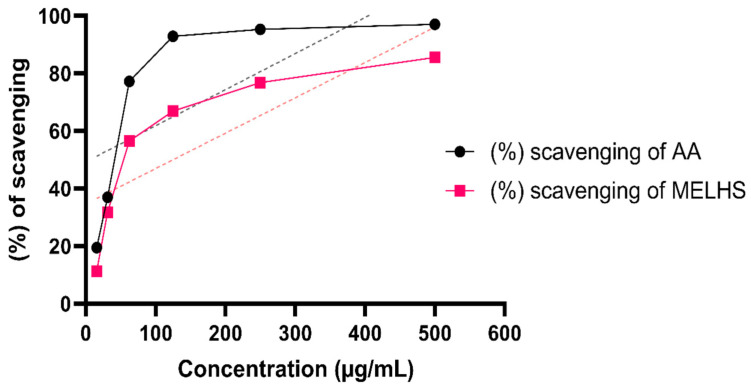
Percentage of radical scavenging activities exhibited by the 1,1-diphenyl-2-picrylhydrazyl (DPPH) assay of the methanol extract of *L. hyalina* stems (MELHS) and standard drug ascorbic acid (AA) at different concentrations.

Through the DPPH free radical scavenging qualitative assay, the antioxidant properties of MELHS were estimated. Figure 1 demonstrates that MELHS has a good antioxidant potency compared with the standard application of ascorbic acid. As can be seen, the scavenging capacity increased compared with the increased concentration of ascorbic acid. The sample extract exhibited a maximum scavenging capacity (85.52%) at a 500 μg/mL concentration, while the standard demonstrated a maximum scavenging capacity of 97.05% at the same concentration. The IC_50_ values for ascorbic acid and MELHS (5.93 and 125.16 μg/mL, respectively) were estimated via the linear regression equation. Here, the linear regression equation was y = 0.1518x + 28.697, with a correlation coefficient (R^2^) of 0.8278.

#### 3.2.2. Reducing Power Assay

**Figure 2:**
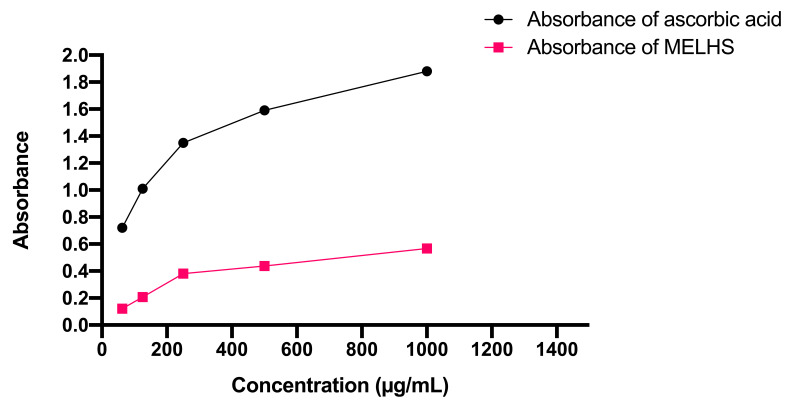
Reducing power of MELHS and standard drug ascorbic acid (AA) at different concentrations.

An antioxidant ability assessment test is summarized in Figure 2 for different doses of ascorbic acid and MELHS. It can be seen that, as the mass concentrations of extracts increase, so does the reducing power of both the standard and sample extracts. The highest absorbance of MELHS was 0.568 at a 1000 μg/mL concentration, whereas at the same density, the absorbance of ascorbic acid was 1.88. 

#### 3.2.3. Total Phenolic and Flavonoid Contents

A quantitative study of the antioxidant-related total phenolic and flavonoid content of MELHS was carried out using a regression equation (for total phenolic, the regression equation was y = 0.0039x + 0.033 and for flavonoid, it was y = 0.0102x − 0.0637). The total antioxidant-related phenolic and flavonoid contents of MELHS were 150.96 ± 1.04 mg GAE/g LH and 47.32 ± 0.77 mg QE/g LH, respectively.

### 3.3. Cytotoxic Activity

**Figure 3:**
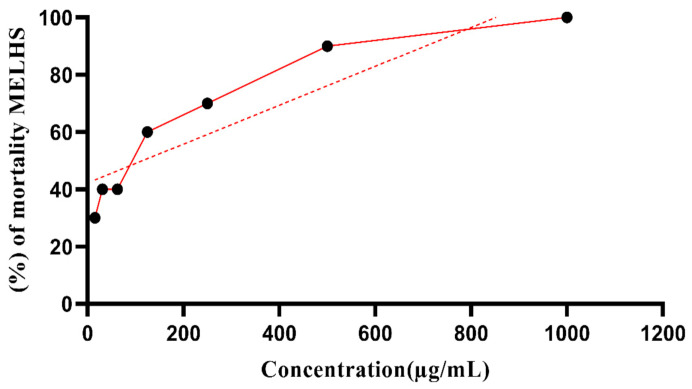
Percentage of mortality of brine shrimp at different concentrations of MELHS.

A brine shrimp cytotoxic assay was conducted to evaluate the cytotoxic potency of MELHS. Figure 3 shows the fatality percentages, as well as the LC_50_ (115.11 μg/mL) value derived using the equation. The average percentage was found to be 61.43% in a dose-dependent model. A 100% mortality rate was observed for a 1000 μg/mL concentration of sample extract, while a 30% mortality rate was observed for a 15.625 μg/mL concentration.

### 3.4. Thrombolytic Activity

**Figure 4:**
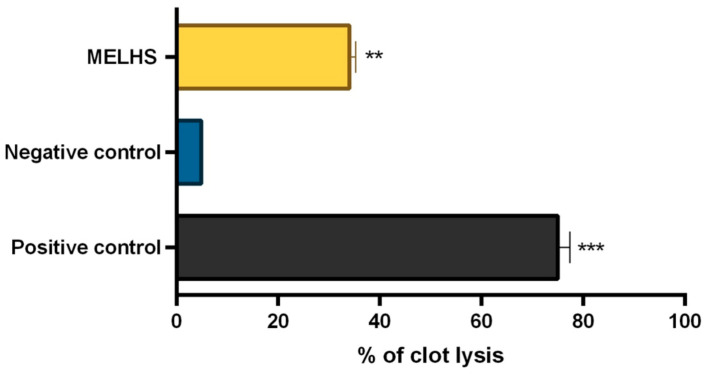
Percentage of clot lysis of MELHS. The results are expressed as the mean ± SEM, where ** *p* < 0.01 and *** *p* < 0.001 are considered statistically significant. The statistical analysis followed by one-way analysis of variance (Dunnett’s test) compared to the negative control (1% Tween-80) using GraphPad Prism version 6.0.

The thrombolytic activity and health status of an adult volunteer are shown in Figure 4. The application of MELHS resulted in a reasonable thrombolytic ability: A 10 mg/mL concentration of sample extract exhibited a 33.98% clot-lysis capability, whereas the positive control (streptokinase) demonstrated a 75% clot-lysis capability. However, the negative control (normal saline) exhibited low clot-lysis capability (4.84%).

### 3.5. Acute Oral Toxicity Test

No behavioral change or mortality was observed during the observation period of 72 h. As a result, a dose of MELHS of up to 2000 mg/kg was recorded as being safe.

### 3.6. Anxiolytic Activity

#### Elevated Plus Maze (EPM)

**Figure 5:**
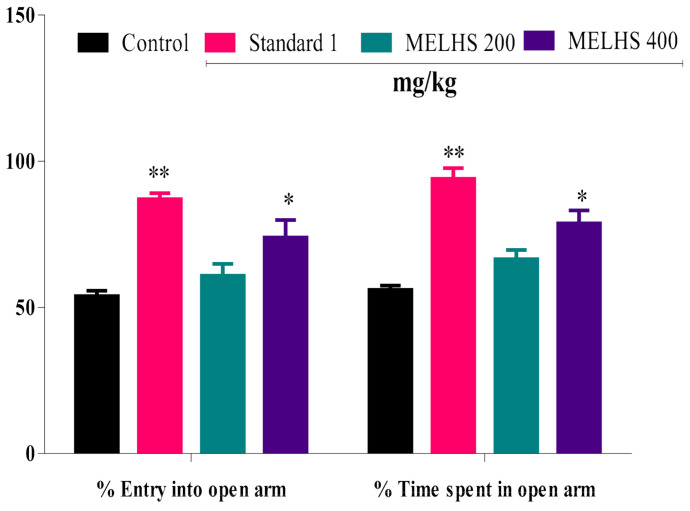
Exploration of the anxiolytic behavior of MELHS and diazepam (standard) in an elevated plus-maze test. The results are expressed as the mean ± SEM, where * *p* < 0.05 and ** *p* < 0.01 are considered statistically significant. The statistical analysis followed by one-way analysis of variance (Dunnett’s test) compared to the negative control (1% Tween-80) using GraphPad Prism version 6.0.

This experiment was conducted to evaluate the anti-anxiety potency of MELHS. The use of MELHS in animals showed promising results. It significantly increased the number of entries into the open arms, as well as the time spent in the open arms, as shown in Figure 5. For the oral 200 mg/kg dose, the percentage of entries into the open arms and the time spent in the open arms were 60.84 ± 4.05 and 66.50 ± 3.14, respectively. In contrast, for the 400 mg/kg dose, the percentage of entries into the open arms and the time spent in the open arms were 73.89 ± 5.97 and 78.77 ± 4.42 (*p* < 0.05), respectively. For the control dose (10 mL/kg), the percentage of entries into the open arms and the time spent in the open arms were 53.86 ± 1.84 and 56.02 ± 1.48, respectively. The diazepam (1 mg/kg) treatment also significantly increased the percentage of entries into the open arm and the time spent in the open arms, producing values of 87.02 ± 2.08 and 94.03 ± 3.63 (*p* < 0.01), respectively.

### 3.7. Antidepressant Activity 

#### 3.7.1. Tail Suspension Test 

**Figure 6:**
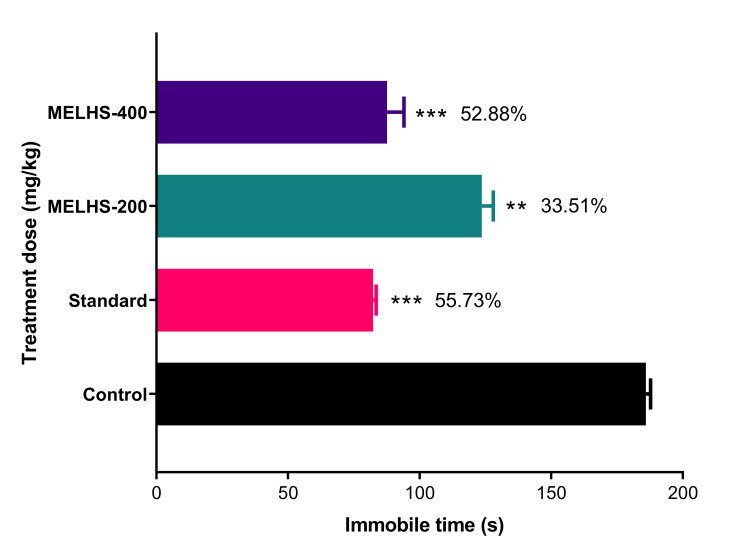
Exploration of the antidepressant activity of MELHS in a tail suspension test in mice. The results are expressed as the mean ± SEM, where ** *p* < 0.01 and *** *p* < 0.001 are considered statistically significant. The statistical analysis followed by one-way analysis of variance (Dunnett’s test) compared to the negative control (1% Tween-80) using GraphPad Prism version 6.0.

In this anti-depression study, the applied dose of MELHS exhibited noteworthy results compared with the standard drug, as shown in Figure 6. The methanol extract showed a dose-dependence tendency. The immobile time of the experimental mice decreased from 123.67 ± 4.26 (33.51%) to 87.67 ± 6.40 s (52.88%) as the doses were increased from 200 to 400 mg/kg, respectively. Both doses presented significant results (*p* < 0.01) compared with the control (186 ± 1.69). The immobile time of fluoxetine (20 mg/kg) was 82.33 ± 1.18 s (55.73%), which was also significant compared with the control (*p* < 0.001). 

#### 3.7.2. Forced Swimming Test 

**Figure 7:**
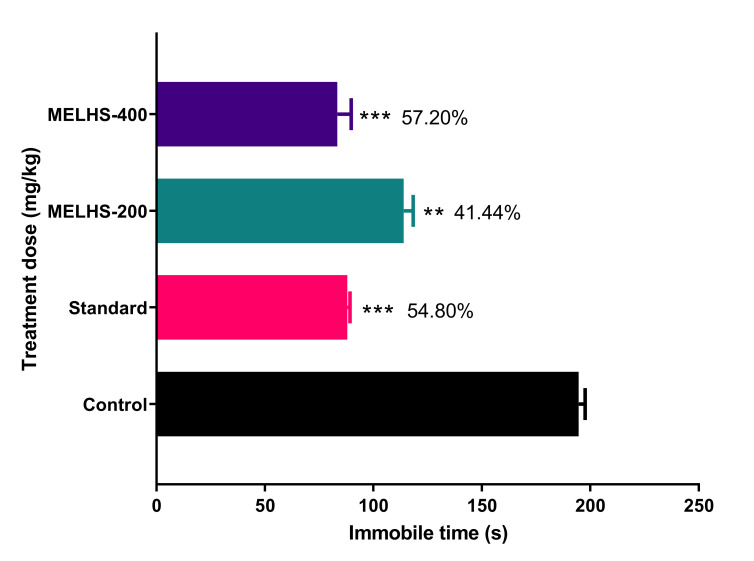
Exploration of the antidepressant activity of MELHS in a forced swimming test in mice. The results are expressed as the mean ± SEM, where ** *p* < 0.01 and *** *p* < 0.001 are considered statistically significant. The statistical analysis followed by one-way analysis of variance (Dunnett’s test) compared to the negative control (1% Tween-80) using GraphPad Prism version 6.0.

The methanol extract revealed anti-depressant-like bioactivity in mice in the forced swimming tests shown in Figure 7. Showing a dose-dependence tendency, the extract at 200 and 400 mg/kg significantly decreased the immobility time to 114 ± 4.36 (41.44%) and 83.33 ± 6.39 s (57.20%), respectively, in comparison with the control group (194.67 ± 2.91 s) (*p* < 0.01). The immobility time for the reference drug fluoxetine (20 mg/kg) also significantly reduced the immobility time to 88 ± 1.15 s (54.80%), (*p* < 0.001). 

## 4. Discussion

Medicinal plants play a significant role in medical science by modulating various human dysfunctions. From the beginning of civilization, human beings have depended on medicinal plants for curing their bodily disorders. As a potential source of biologically active compounds, medicinal plants may definitely be considered superior [37,38]. They have traditionally been used for curing various kinds of diseases [39]. Several chemicals derived from plants serve as medicinal agents, offering a range of biological activity: Antioxidant, cytotoxicity, thrombolytic, anxiolytic, antidepressant, neuroprotective, hepatoprotective, etc. [40,41,42,43,44,45]. In qualitative and quantitative phytochemical analyses, MELHS has demonstrated various types of phytochemical groups. The phytochemical analysis in this study confirmed the presence of alkaloids, carbohydrates, flavonoids, saponin, tannin, triterpene, phenol, quinone, cardiac glycoside, and coumarin, as well as an extensive range of polyphenolic compounds (Table 1). It might therefore be possible that such phytochemicals were responsible for the DPPH scavenging activity and ferric-reducing capacity of the MELHS plant extract [46,47,48,49,50,51].

The brine shrimp lethality bioassay is a more suitable method for observing the biological properties of natural products [52]. It is a fast, cheap, and simple biological assay for evaluating the plant extract toxicity, which in many cases, connects reasonably with cytotoxic and antitumor or anticancer activities [53,54]. In this experiment, the brine shrimp bioassay was used to estimate the lethality of crude extracts of MELHS. An estimation of the toxicity of plant extracts is obligatory when determining their safety as a treatment, in order to detect both the inherent toxicity of the plant and the outcome of an acute overdose. This study has helped gauge the biological reaction to the natural plant extract [55,56]. Furthermore, it has helped to established the dosages that can be administered to animal models [57]. In the calculation of the overall toxicity using brine shrimp, the maximum fatality occurred at a 1000 μg/mL concentration, while the minimum fatality occurred at a 10 μg/mL concentration. The cytotoxic properties were measured as weak in the range 500 ≥ LC_50_ ≤ 1000 μg/mL, moderate in the range 100 ≥ LC_50_ ≤ 500 μg/mL, strong in the range 0 > LC_50_ < 100 μg/mL, and nontoxic in the ranged LC_50_ > 1000 μg/mL [58]. In this study, the rate of mortality was directly proportional to the concentration of the plant extract of MELHS, with LC_50_ of 115.11 μg/mL, which is moderately cytotoxic. The cytotoxic bioactivity of MELHS may be due to the existence of anti-tumor components, as well as the presence of flavonoid contents, in the MELHS plant extract [59].

Thrombolysis is the dissolution of hazardous clots in blood vessels for promoting blood flow and averting injury to tissues and organs [60]. This blood accumulation leads to an internal hemorrhage, which may threaten vital organs such as the brain, heart, kidneys, etc. Thrombolytic agents such as streptokinase are used to dissolve blood clots [61]. Generally, to liquefy blood coagulates, a thrombolytic mediator triggers the plasminogen that forms plasmin, which acts as a cleavage factor [62,63,64]. This is a proteolytic enzyme that is capable of preventing cross-links between fibrin particles. Various plant sources such as herbs, leaves, fruits, and seed may have anti-clot, anti-platelet, and fibrinolytic bio-properties. In this study, it was found that the capacity for the clot lysis of MELHS was 33.98% (*p* < 0.01) at a 10 mg/mL concentration, which was significant compared with the negative control, while the streptokinase and negative control (normal saline) showed a capability for clot lysis of 75% and 4.84%, respectively. The thrombolytic properties of MELHS may arise from the presence of flavonoids in the methanol extract [60].

The elevated plus-maze (EPM) test is a behavioral experiment used to measure anxiety in mice, based on the animals’ aversion to open spaces. In this study, entry into open arms and closed arms was available to the mice. In the EPM test, the plant extract’s anxiolytic activity caused a greater number of entries and more time spent in open arms than in closed arms [61]. Moreover, the 400 mg/kg dose significantly increased the percentage of entries into open arms and the time spent in open arms to 73.89 ± 5.97 and 78.77 ± 4.42 (*p* < 0.05), respectively. For the standard candidate (diazepam), the respective percentages were 87.02 ± 2.08 and 94.03 ± 3.63 (*p* < 0.01). MELHS can therefore be said to produce a significant anxiolytic effect, probably due to the presence of phenolic compounds [62,63].

Depression is a mood-altering disorder. Its symptoms include feelings of sadness, loss, or anger that interfere with a person’s everyday activities. Its many causes include stress, long-term anxiety, and several chronic disorders [64,65,66,67]. It has also been reported that depressed people have increased oxidative stress and reduced anti-oxidant defenses [68]. To investigate the antidepressant properties of MELHS, the current study conducted forced swimming and tail suspension tests. In these tests, mice were compelled to swim in water or allowed to hang by their tails, in each case creating a situation of despair. In such cases, lower immobility times indicate higher rates of anti-depressant activity [69,70]. In TST and FST, the maximum immobility times of mice were 87.67 ± 6.40 s (52.88%), (*p* < 0.001) and 83.33 ± 6.39 s (57.20%), (*p* < 0.001), respectively, for a 400 mg/kg oral dose, against the positive control (20 mg/kg fluoxetine), whose immobility times were 82.33 ± 1.18 s (55.73%), (in TST) and 88 ± 1.15 s (54.80%), (*p* < 0.001) (in FST). The anti-depressant results of MELHS are therefore close to the values for the standard drug, indicating very good anti-depressant activity. A number of previous studies have suggested that the presence of total phenols and flavonoids may be responsible for this antidepressant effect [71,72].

## 5. Conclusions

The collected pharmacological findings indicate the strong potential of this plant. This study reveals that the extract is a potential source of antioxidants. It also argues that MELHS offers noteworthy thrombolytic activity and moderate cytotoxic activity. Additionally, the plant extract may have potential applications in the treatment of psychiatric conditions such as anxiety and depression, probably due to the presence of phenol and flavonoids in the plant extract. Overall, this plant can be recognized as a possible candidate for the creation of a new drug compound with numerous pharmacological applications. Further extensive studies are still required to identify the secondary metabolites of the plant responsible for the biological activities observed, and also to determine the mechanism behind its therapeutic activities.

## Figures and Tables

**Table 1:** Result of the phytochemical screening of *Lepidagathis* *hyalina* Nees stems.

Phytochemicals	Type of Test	Appearance	Results
Alkaloids	Mayer’s test	Yellow color	++
	Wagner test	A reddish brown color	++
Carbohydrates	Molisch’s test	Reddish color ring form	++
Glycosides	Shinoda test	No deep red color	−
Reducing sugar	Fehling’s test	Red precipitate form	++
	Benedict’s test	Reddish color precipitate form	++
Flavonoids	Lead acetate test	Florescence yellow color form	++
Saponins	Froth test	Persistent forth for one hour	+
Tannin	FeCl_3_ test	Brownish green appears	+
Sterols	Liebermann–Burchard test	No layer form	−
Triterpene	Salkowski test	Reddish color form	+
Resin	FeCl_3_ test	No precipitation	−
Phenol	FeCl_3_ test	Violet color form	++
Quinones	HCl test	Yellow color present	+
Cardiac Glycoside	Legal test	Brown color	+
Coumarins	Ammonia test	Green color form	++
Cholesterols	General test	No red rose color	−
Terpenoids	Salkowski’s test	Reddish brown not form	−

Here, ++, highly present; +, moderately present; and −, absent.

## Data Availability

Available data are presented in the manuscript.

## Abbreviations

MELHS, methanol extract of *Lepidagathis hylina* Nees stems; DPPH, 1,1-diphenyl-2-picrylhydrazyl; FCR, Folin–Ciocalteau reagent; UV, ultra-violet; BW, body weight; TPC, total phenol content; TFC, total flavonoid content; EPM, elevated plus maze; TST, tail suspension test; FST, forced swimming test; SEM, standard error mean; p.o., per oral; i.p., intraperitoneal.
